# Multidisciplinary Pain Management for Pediatric Patients with Acute and Chronic Pain: A Foundational Treatment Approach When Prescribing Opioids

**DOI:** 10.3390/children6020033

**Published:** 2019-02-21

**Authors:** Anava A. Wren, Alexandra C. Ross, Genevieve D’Souza, Christina Almgren, Amanda Feinstein, Amanda Marshall, Brenda Golianu

**Affiliations:** 1 Department of Pediatrics, Division of Pediatric Gastroenterology, Hepatology and Nutrition, Stanford University, Palo Alto, CA 94305, USA; awren2@stanford.edu; 2 Department of Pediatrics, Child and Adolescent Headache Program, University of California San Francisco, San Francisco, CA 94158, USA; alexandra.ross2@ucsf.edu; 3 Department of Anesthesiology Perioperative and Pain Medicine, Stanford University, Palo Alto, CA 94305, USA; gdsouza@stanford.edu (G.D); calmgren@stanfordchildrens.org (C.A.); abfein@stanford.edu (A.F.); 4 Centre for Clinical Brain Sciences, University of Edinburgh, Edinburgh EH16 4SB, UK; a.marshall-15@sms.ed.ac.uk

**Keywords:** multidisciplinary pain management strategies, opioid reduction therapy, non-pharmacological therapy, cognitive behavioral therapy, hypnosis, mindfulness-based stress reduction, acupuncture, pain rehabilitation

## Abstract

Opioid therapy is the cornerstone of treatment for acute procedural and postoperative pain and is regularly prescribed for severe and debilitating chronic pain conditions. Although beneficial for many patients, opioid therapy may have side effects, limited efficacy, and potential negative outcomes. Multidisciplinary pain management treatments incorporating pharmacological and integrative non-pharmacological therapies have been shown to be effective in acute and chronic pain management for pediatric populations. A multidisciplinary approach can also benefit psychological functioning and quality of life, and may have the potential to reduce reliance on opioids. The aims of this paper are to: (1) provide a brief overview of a multidisciplinary pain management approach for pediatric patients with acute and chronic pain, (2) highlight the mechanisms of action and evidence base of commonly utilized integrative non-pharmacological therapies in pediatric multidisciplinary pain management, and (3) explore the opioid sparing effects of multidisciplinary treatment for pediatric pain.

## 1. Introduction

Acute and chronic pain are common and often debilitating problems among both pediatric and adult populations according to the Institute of Medicine statement on Relieving Pain in America. The International Association of the Study of Pain (IASP) defines pain as an unpleasant sensory and emotional experience associated with actual or potential tissue damage or described in terms of such damage [1]. Acute pain is the expected physiological response to a noxious chemical, thermal, or mechanical stimulus, and usually accompanies surgery, traumatic injury, tissue damage, or inflammatory processes. It is self-limiting and typically resolves over days to weeks, but it can persist longer as healing occurs [2]. Chronic pain is defined as intractable pain that exists for three or more months despite adequate treatment [1,3].

Opioid therapy is the cornerstone of treatment for acute procedural and postoperative pain in pediatric populations, and is also regularly prescribed for severe and debilitating chronic pain conditions [4]. Despite the benefits of opioids for pain management, opioids have been associated with a range of side effects including respiratory depression, constipation, cognitive dysfunction, and psychiatric comorbidities [5,6,7,8]. Persistent opioid use is also associated with physical tolerance, dependence, and addiction [9], as well as heightened pain sensitization [10].

Given the potential for negative outcomes related to opioid use, there have been increased efforts to use multidisciplinary analgesia treatments (i.e., the concurrent use of treatment provided by practitioners from different disciplines) [11]. Combining pharmacological and integrative non-pharmacological therapies, which operate via different modes of action, has been shown to decrease opioid use and related adverse side effects in the perioperative period, as well as improve acute pain symptoms and emotional wellbeing [12]. When acute pain transforms to chronic pain, the consideration of multidisciplinary care may become even more central in facilitating patient comfort, decreasing reliance on opioid therapy, and improving functionality. Of note, in 2016, the CDC published recommended guidelines for prescribing opioids for adults with non-malignant chronic pain in the US stating [13]:

“*Nonpharmacologic therapy and nonopioid pharmacologic therapy are preferred for chronic pain. Clinicians should consider opioid therapy only if expected benefits for both pain and function are anticipated to outweigh risks to the patient. If opioids are used, they should be combined with nonpharmacologic therapy and nonopioid pharmacologic therapy, as appropriate*.”

For patients 18 years and younger, there do not yet exist specific consensus guidelines on prescribing opioids for chronic pain [4,14,15]. Pediatric practitioners are advised to use their best judgment when using opioids after appropriate use of nonopioid alternatives. Opioids are not usually indicated as a first line therapy for primary pain disorders [16]. However, current evidence suggests that opioid prescriptions should not be curtailed for moderately to severely painful conditions [17,18].

### Aims

Overall, multidisciplinary treatment is important to consider in the treatment of both acute and chronic pain. The aims of this paper are to: (1) provide a brief overview of a multidisciplinary pain management approach for pediatric patients with acute and chronic pain, (2) highlight the mechanisms of action and evidence base of commonly utilized integrative non-pharmacological therapies in pediatric multidisciplinary pain management, and (3) explore the opioid sparing effects of multidisciplinary treatments for pediatric pain.

This article will discuss the evidence base of several integrative non-pharmacological treatments (cognitive behavioral therapy, mindfulness, medical hypnosis, acupuncture) for the general pediatric practitioner to consider as part of a multidisciplinary treatment plan in the management of complex pain conditions. The therapies discussed were chosen as they are among the most frequently utilized integrative non-pharmacological therapies in pediatric pain clinics [19]. The aim of this review is not to be exhaustive of each integrative non-pharmacological therapy, but rather to provide an overview of the specific therapy, briefly review mechanisms of action as currently understood, and provide relevant research on the topic. While a systematic review of the aforementioned integrative non-pharmacological therapies is outside the scope of this current paper (for several excellent and relevant systematic reviews, please see Fisher et al., 2018 [20] and Kamper et al., 2015 [21]), the overarching goal of this paper is to educate the general pediatric practitioner in evidence-based integrative non-pharmacological therapies included in multidisciplinary pain management to optimize care for youth with acute and chronic pain conditions.

Throughout this paper, we review both pediatric and adult literature. A search was performed using Pubmed, Ovid, Embase, Prospero, Medline, and Cochrane Database on the following topics in both pediatric and adult literature from 2000 to 2018 (see Table 1). Inclusion criteria were: pediatric chronic pain and each of the following: multidisciplinary treatment, cognitive behavioral therapy, mindfulness, hypnosis, acupuncture, and pain rehabilitation. Opioids and opioid reduction therapy were also used as inclusion criteria with each modality. Exclusion criteria were: pediatric procedural pain, acute pediatric pain, pharmacological management of pediatric pain, and regional management of pediatric pain.

Regarding article selection criteria, studies were screened and identified by two authors (AW and AR for Cognitive Behavioral Therapy (CBT), mindfulness and hypnosis; CA and GD for multidisciplinary pain management, acupuncture and rehabilitation), and when a consensus could not be reached regarding the inclusion/exclusion of an article in this review BG mediated and made a final decision. The research team prioritized systematic reviews, non-systematic reviews, and randomized controlled studies of these therapies in pediatric pain. Where these were not available, we identified pilot studies in pediatric pain populations to provide the reader with the current state of science and preliminary evidence for a given integrative non-pharmacological therapy. Pediatric pain articles were supplemented with adult articles where systematic reviews were not available, and where studies investigating mechanisms of action were well elucidated in the adult literature and not studied in pediatrics. Of note, adult studies exploring potential mechanisms of action for integrative non-pharmacological therapies are not meant to imply that adult mechanisms are the same as pediatric mechanisms of action, but rather to inform the reader of the current evidence base of this emerging field of research. Due to limitations of space, the use of these interventions to address specific procedural and/or acute pain is not discussed.

## 2. Multidisciplinary Pain Management Overview

**Figure 1:**
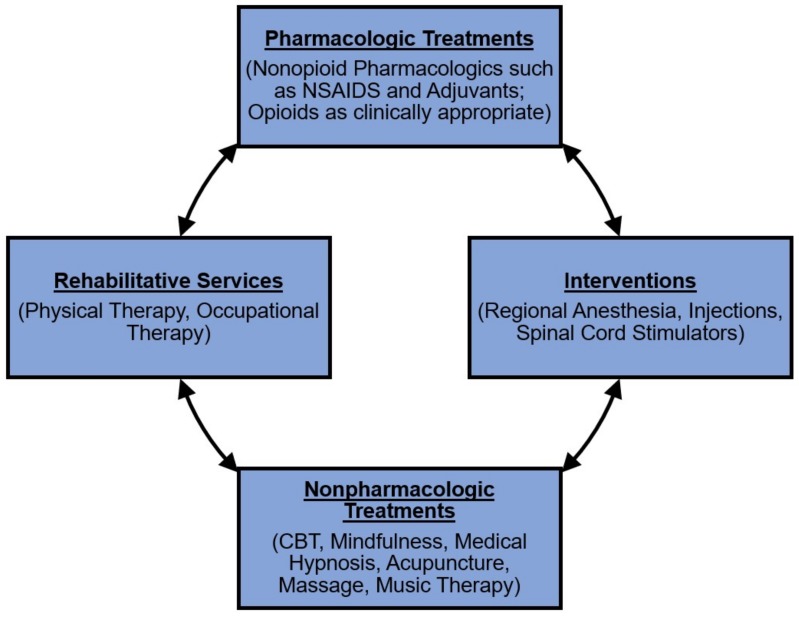
Multidisciplinary pain management treatment: key components in acute and chronic pain management. This figure displays the key treatment components in multidisciplinary treatment for both acute and chronic pain. In the acute setting, in addition to reduction of pain, the efficacy of multidisciplinary treatments is often measured by reduction in needed opioid doses to achieve comfort, while in the setting of chronic pain, the improvements obtained through a multidisciplinary approach are often measured by improvements in function. As is clinically appropriate, in both settings, pharmacologic treatments are combined with regional interventions [26], integrative non-pharmacological techniques, and rehabilitative services as is clinically appropriate to support pain management and improve patients’ pain symptoms, functioning and quality of life. Multidisciplinary analgesia treatment aims to ensure patient comfort and wellbeing, while at the same time potentially decreasing the need for opioid use in pediatric populations [25].

Evidence suggests that multidisciplinary analgesia treatments incorporating nonopioid pharmacological and integrative non-pharmacological therapies can be effective for both acute and chronic pain management, and can improve patients’ quality of life and general wellbeing [13,21,22,23,24]. Integrative non-pharmacological therapies include modalities such as cognitive behavioral therapy (CBT), mindfulness, medical hypnosis, acupuncture, massage, and music therapy [25]. See Figure 1 for an overview of some elements of multidisciplinary pain management treatment.

### 2.1. Acute Multidisciplinary Pain Management

**Figure 2:**
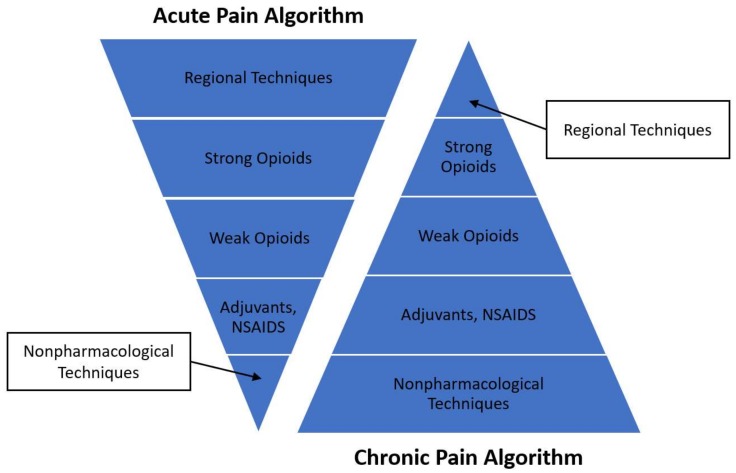
Multidisciplinary pain management: acute and chronic pain algorithms. In acute pain algorithms, the initial treatment begins with regional techniques or intravenous analgesia as a mainstay of therapy. As acute pain improves, therapies are then transitioned as appropriate to varying strengths of PO opioid medications, to adjuvants/NSAIDS, and ultimately integrative non-pharmacological strategies. For situations where severe pain is anticipated, adjuvants and integrative non-pharmacological strategies may be added on at the beginning of treatment, as an opioid sparing strategy, and to increase patient comfort. In chronic pain algorithms, treatment is delivered in the reverse order, beginning with integrative non-pharmacological techniques, then moving to adjuvants, and ultimately progressing to various strengths of opioids and regional techniques and stimulators as clinically appropriate.

Multimodal treatment is defined as “the concurrent use of separate therapeutic interventions with different mechanisms of action within one discipline aimed at different pain mechanisms” [11]. This treatment approach involving multiple medications for pain management is considered optimal in the setting of acute pain (see Figure 2), where the primary goal is immediate analgesia sufficient to allow for the recovery from medical treatments/procedures with minimal side effects.

While acute pain management has historically emphasized the use of opioids, multimodal analgesia treatment incorporates opioid-sparing adjuvants targeting specific aspects of the nociceptive and neuropathic pain physiology. Nociceptive analgesics include acetaminophen, NSAIDS, and glucocorticoids. Neuropathic analgesics commonly include gabapentinoids, lidocaine, ketamine, and alpha 2 agonists. Regional anesthesia and injection of local anesthetics are also regularly utilized to allow targeted analgesia to a specific surgical area. These agents provide safe and effective analgesia and sedation and may help reduce the need for opioid therapies [27,28,29].

Acute pharmacological interventions can be enhanced by the assistance of integrative non-pharmacological interventions (i.e., multidisciplinary treatment). In treatment of pediatric acute pain, the integration of a psychologist or child life specialist into the treatment team can support pain management efforts in the perioperative period by educating the patient in an age appropriate manner about medical procedures, a pain management plan, and implementing behavioral pain management interventions (e.g., distraction, play, active relaxation training) to reduce post-operative pain and anxiety [30]. An acupuncturist or massage therapist can also support acute pediatric pain management following medical procedures and surgeries [31,32]. Incorporating integrative non-pharmacological therapies into multidisciplinary analgesia treatments for acute pediatric pain has been shown to reduce perioperative anxiety and procedural pain [33,34], and thus has the potential to reduce pediatric patients’ reliance on pharmacological interventions such as opioids and benzodiazepines. Preliminary evidence supports this notion, demonstrating that even in an acute medical setting such as the management of perioperative pain following a minimally invasive surgery for Pectus Excavatum repair (Nuss procedure) in adolescents, the addition of an integrative non-pharmacological therapy (in this example, self-hypnosis instruction preoperatively) can reduce pain, postoperative opioid use, and even length of hospital stay [35].

### 2.2. Chronic Multidisciplinary Pain Management

In the management of chronic pain, the emphasis shifts from immediate analgesia to extended pain management services and facilitation of function across domains (e.g., performance of activities of daily living). A multidisciplinary approach to chronic pain management (see Figure 2) has therefore increasingly become the standard of care in pediatric pain management settings. Chronic pain treatment places a central emphasis on integrative non-pharmacological techniques, minimizing side effects, and ideally providing the child skills they can use to more independently manage their symptoms.

Oftentimes, in pain management of complex patients, the utility of integrative non-pharmacological therapies is discussed only when all pharmaceutical therapy options have been exhausted. By then, the patient may begin to feel that medical care is somehow being limited or withdrawn, or that using integrative non-pharmacological therapies indicates that the “pain is in their head”. This can lead to patients and families feeling resistant to learning and incorporating these essential integrative non-pharmacological pain management tools. In multidisciplinary chronic pain management, integrative non-pharmacological therapies are incorporated in early phases of treatment, along with appropriate pharmacological interventions to provide optimal care for complex pediatric patients.

Pharmacological interventions in this setting often include analgesics such as acetaminophen and nonsteroidal agents, gabapentinoids, clonidine, tricyclic antidepressants, and where appropriate serotonin-norepinephrine reuptake inhibitors. Another novel adjuvant therapy that deserves mention as a possible opioid sparing agent, cannabidiol (CBD), is gaining popularity in multimodal pain management. CBD is the nonpsychoactive cannabinoid identified in cannabis, while tetrahydrocannabinol or THC is responsible for the hallucinogenic side effects of cannabis [36]. Further research is needed to examine the efficacy and side effect profile of CBD in chronic pain management, and to elucidate side effects and risks.

Opioids are typically minimized in chronic pain management, as the analgesic benefits of opioids are often offset by their long-term negative side effects such as constipation, nausea, vomiting, and sedation, as well as the risk of tolerance, dependence, opioid-induced hyperalgesia, and addiction [5,6]. If opioids are deemed clinically necessary, weak opioids like Tramadol or Hydrocodone can be used in the initial treatment phase but stronger opioids may be needed with recalcitrant pain. Regional anesthesia also may play less of a role, as the duration of the local anesthetic and length of time a catheter can be safely inserted may not provide longer-term analgesic benefit.

## 3. Integrative Non-Pharmacological Therapies in Pediatric Multidisciplinary Pain Management

Incorporating integrative non-pharmacological interventions into pediatric multidisciplinary chronic pain management has been demonstrated to be feasible and effective and has become the gold standard of care [27,37,38,39]. These techniques aim to modulate psychological factors, improve coping abilities, and enhance emotional well-being. Specifically, these therapies are thought to target: cognitions (e.g., modifying the child’s anxious thoughts related to distressing physical sensations), emotions (e.g., teaching the child emotion regulation strategies and distress tolerance skills to support reductions in negative emotional/somatic symptoms), behaviors (e.g., decreasing pain avoidance behaviors and increasing the use of coping skills), and sensory experiences (e.g., directly targeting pain perceptions through enhanced nervous system inhibitory processes) [37,40]. In contrast to short-term pharmacological management, which provide patients transitory benefit, integrative non-pharmacological techniques can lead to long-term results via changes in neural circuits that regulate habits, affect, and cognitive pain responses [41]. Directly addressing cognitive-affective processes may increase youths’ coping repertoire of strategies to manage pain (short-term and long-term), regulate mood, improve psychological well-being and resiliency, and potentially buffer against opioid misuse.

For the purpose of this review, we focus on four frequently utilized interventions in U.S. pain clinics, which include three psychologically based interventions: cognitive behavioral therapy, mindfulness, and medical hypnosis. Acupuncture, an integrative medicine approach widely used in pain management clinics, is additionally reviewed [39,42,43,44,45]. Rehabilitative therapies, including physical and occupational therapy (PT, OT), are also an important part of the treatment of chronic pain and are regularly integrated into care to support pain management and increase function [39,46,47,48,49]. Of note, rehabilitative therapies are uniquely dependent on the condition being treated (e.g., back pain, ankle sprain, complex regional pain syndrome), and as such, a comprehensive review of PT and OT in pediatric pain was deemed outside the scope of this paper. Overall, there is growing evidence that integrative non-pharmacological techniques can provide lasting pain management benefits, [37] have the potential to decrease reliance on opioids [41], and may minimize side effects [50]. Recent evidence also suggests that interdisciplinary pain management programs can even support effective opioid weaning among youth with chronic pain [14,51]. The importance of integrating non-pharmacologic strategies into the care of youth with acute and chronic pain will be explored below. See Appendix A for a summary of select studies reviewed.

Lastly, it is important to highlight that the integrative non-pharmacological treatments reviewed below often require significant commitment and effort from both children and families, and while frequently effective, improvements typically occur over time. Some barriers to implementation include: patient and family “buy-in” to the proposed treatment; work, family, and scheduling demands; financial challenges; accessibility of local resources (e.g., no accessible providers trained in a particular treatment modality); and the general burden of care. Awareness, sensitivity, and attention to such issues are required on the part of providers to best support patients and families in structuring their multidisciplinary treatment plan.

### 3.1. Cognitive Behavioral Therapy

Cognitive behavioral therapy (CBT) is a form of psychotherapy that has empirical support across a wide range of child, adolescent, and adult populations and is considered an evidence-based intervention in multidisciplinary chronic pain management [52,53]. CBT is based on the premise that thoughts, emotions, and behaviors are closely connected, and how one perceives a situation can significantly influence emotional, behavioral, and physiological responses [54]. When considering CBT for the treatment of chronic pain, protocols often consist of behavioral strategies that encourage engagement in normal daily activities (e.g., activity pacing to increase functioning across domains), cognitive techniques that support challenging and reframing negative “self-talk” statements (e.g., acknowledging catastrophic pain-related thoughts—“This pain is ruining my life”—and using evidence to determine more helpful responses—“This pain is bothersome, but I can handle it and go forward”), and self-regulation skills that help decrease physiological arousal and increase relaxation and wellbeing (e.g., diaphragmatic breathing, progressive muscle relaxation). Acceptance and Commitment Therapy (ACT), an extension of CBT, aims to improve functioning by increasing the ability to act effectively in the presence of pain and distress (i.e., psychological flexibility) [55]. CBT strategies are effective with a wide age range when adapted to the specific developmental level of the child or adolescent [56].

Of note, in the adult literature, neuroimaging data suggest that cognitive reappraisal of pain, a central CBT strategy for patients with chronic pain, may impact pain experiences through top-down processes resulting in increased prefrontal cortex gray matter [57]. CBT has also been linked to increased activation in the ventrolateral prefrontal and lateral orbitofrontal cortices, providing additional support for the role of a cortical control mechanism in CBT [58]. Additionally, CBT for chronic pain has led to changes in somatosensory cortices, potentially reflecting alterations in the perception of noxious signals (i.e., adaptive responses to pain signals) [57]. 

#### 3.1.1. Evidence for CBT and Pain Management

There is a strong evidence base for the effectiveness of CBT among adults with chronic pain. A meta-analysis of 25 randomized controlled trials of CBT for adults with chronic pain noted significant changes in patients’ reported pain experiences, cognitive coping, and behavioral expressions of pain as compared to wait list controls [59].

CBT is also the most well-established psychological intervention among youth with chronic pain [20,60]. A growing evidence base of systematic reviews and meta-analyses have documented that behavioral and cognitive behavioral interventions can produce a medium to large effect on pain outcomes and small effect on disability in chronic pain populations (e.g., headache, abdominal pain). [37,61,62]. In one strong meta-analytic review of randomized controlled trials of psychological therapies for the management of chronic pain in children and adolescents, Palermo and colleagues noted positive effects of CBT on clinically significant pain reduction, with an odds ratio of 4.13 across 9 studies with 406 participants [61]. Recent research is also beginning to explore various models of treatment delivery for CBT for chronic pain including online modules [63] and group-based formats [64,65]. As compared to other evidence-based psychological therapies, CBT is currently the dominant therapeutic model implemented in both medical and non-medical settings and is viewed as well accepted by patients and providers alike [60].

#### 3.1.2. CBT Summary

In sum, while additional research is needed (e.g., larger RCTs with varying treatment delivery modes and monitoring of longer-term follow-up), a growing body of literature demonstrates the efficacy of CBT protocols for children, teenagers, and adults with chronic pain. This evidence-based treatment is well accepted by patients and providers and is increasingly available to pediatric patients. Although youth in more remote areas may have limited access to cognitive behavioral interventions for chronic pain, the advent of web-based treatments holds promise for reaching a wider pediatric audience [63]. 

### 3.2. Mindfulness

Mindfulness has been described as the ability to be aware of the present moment and one’s experiences (e.g., thoughts, emotions, body sensations) in a purposeful, non-judgmental manner [66]. Research demonstrates associations between mindfulness, psychological and physical well-being, and improved health outcomes [67,68,69]. Interventions and training programs targeting the cultivation of mindfulness (i.e., mindfulness-based interventions; MBIs) have received increasing attention in pain management in recent years. The primary goal of MBIs is to teach individuals mindfulness via a series of breathing, meditation, and movement exercises, all of which cultivate present-moment, compassionate awareness [66,70]. Bringing attention to the present in this manner aims to serve as a buffer from ruminating on bothersome physical symptoms such as pain, negative cognitions, and perceived stress that can contribute to the experience of pain [71,72,73].

Neuroimaging studies in adults have provided further support for the mechanisms by which mindfulness meditation practice can lead to change. Specifically, regions of the brain associated with sensory processing and the cognitive modulation of pain (e.g., thalamus, insula, anterior cingulate cortex, orbitofrontal cortex) [74,75] have high concentrations of opioid receptors, [75,76] prompting hypotheses that mindfulness practice may modulate the endogenous opioid system [77]. Other neuroimaging research proposes that mindfulness-induced analgesia is due to a more complex process engaging multiple brain networks and neurochemical mechanisms [78].

#### 3.2.1. Evidence for MBIs and Pain Management

An increasing body of literature has demonstrated that mindfulness interventions are feasible and efficacious in adult pain populations. Systematic reviews and meta-analyses have documented that mindfulness-based protocols can significantly improve pain outcomes (e.g., pain severity, interference, and sensitivity), psychological distress (e.g., depression), and quality of life among adults [79,80,81].

In pediatric populations, pilot studies have demonstrated initial feasibility and acceptability of mindfulness-based interventions for children and adolescents experiencing a range of chronic pain conditions (e.g., musculoskeletal, neuropathic, headache, abdominal pain) [82,83,84]. These mindfulness protocols have also garnered preliminary empirical support for their efficacy in improving pain-related outcomes such as fibromyalgia symptoms, functional disability, cortisol levels, pain acceptance, and emotional distress among youth with pain symptoms [82,83,84,85,86]. The existing research is promising. For example, in one pilot randomized trial of a mindfulness-based intervention for female adolescents with chronic pain, patients reported improvements in coping with pain, and reductions in pre- and post-mindfulness session salivary cortisol levels (*p* < 0.001) [83]. The quality of this research; however, has varied from randomized controlled trials to pilot studies and is limited in generalizability by small sample sizes, lack of a control groups, and lack of random assignment [83,86,87,88].

Given the more robust body of literature supporting the use of mindfulness interventions with youth to address symptoms of anxiety and depression [89], MBIs have become increasingly popular and available in recent years. Many schools are beginning to implement skills-based learning for stress management, including mindfulness, bringing developmentally appropriate adaptations (e.g., teaching is varied and interactive) of the intervention directly to groups of children with little cost [90]. A recent well controlled sizable RCT compared the application of mindfulness-based stress reduction (MBSR) to a health education control group in 300 5th- to 8th-grade students in an urban public school setting and found significantly lower levels of somatization, depression, negative affect, negative coping, rumination, self-hostility, and post-traumatic symptom severity (all *p* < 0.05) as compared to control [91]. Attention to cultural and developmental aspects of the child and family are important in the successful implementation of these therapies as well. There is also increasing availability of mindfulness-based self-administered electronic resources that may help bring this intervention to the most underserved areas. While free or low cost and easily accessible, there is unfortunately little evidence yet available on the efficacy of these electronic resources [92].

#### 3.2.2. Mindfulness Summary

Substantive findings support the use of mindfulness interventions among adults with chronic pain. While preliminary research among pediatric pain populations demonstrates feasibility and acceptability of MBI’s among youth, further research to investigate adaptations of mindfulness protocols for children and adolescents with chronic pain is needed. Clinical judgment is recommended until published research can better guide the use of MBIs among youth with pain. That being said, mindfulness interventions may be low cost or free adjunctive treatments that have fewer side effects as compared to pharmacologic interventions.

### 3.3. Hypnosis

Hypnosis is another integrative non-pharmacological treatment increasingly used in multidisciplinary pain management. Hypnosis has been defined as a state of attentive and receptive concentration generating changes in individuals’ experiences of themselves and their environment [93,94]. While hypnosis usually (but not always) involves relaxation, [95] this treatment modality differs from mindfulness meditation in the thoughtful and deliberate use of therapeutic suggestions to facilitate change in a targeted domain (in the case of hypnotic analgesia, the patient’s sensory experience) [40].

Similar to the impact of opioids on the brain, hypnotic analgesia is hypothesized to enhance nervous system inhibitory processes that attenuate pain experiences [96] through a variety of brain areas involved in pain processing (for a detailed review, see Jensen and Patterson, 2015) [97]. Neuroimaging studies in adults conducted during hypnotic experiences have noted significant signal changes associated with sensation and perception (e.g., primary somatosensory cortex, thalamus, insula), as well as in sensorimotor integration pain systems (e.g., supplementary motor cortex) [97,98,99].

#### 3.3.1. Evidence for Hypnosis and Pain Management

There is a growing evidence base supporting the efficacy of hypnotically induced analgesia, identifying medical hypnosis as a “well established treatment” for pain in adult populations [100,101]. A meta-analysis investigating hypnosis for acute and chronic pain management found that for 75% of patients across studies (18 studies; *n* = 933) hypnosis provided substantial pain relief and was shown to be superior to non-hypnotic psychological interventions [52].

While there is a significantly smaller pediatric research base, extant controlled studies indicate positive results for pain reduction using this treatment modality among pediatric populations. Supportive findings for pain reduction have been noted in youth undergoing surgery, cancer patients, and young people diagnosed with irritable bowel syndrome (IBS) [102,103,104,105]. In one noteworthy randomized controlled trial of hypnosis for children with functional abdominal pain or IBS, hypnosis was demonstrated as highly superior to standard medical therapy in reducing both pain frequency and pain intensity (*p* < 0.001) [104]. Additionally, one recent randomized controlled trial examined self-hypnosis training for 22 self-selected adolescents hospitalized for the Nuss procedure [35]. The results of this study indicated that in addition to better pain control, self-hypnosis training was associated with use of fewer milligrams per hour of morphine equivalents throughout a patient’s five-day hospitalization (*p* = 0.005). A smaller and earlier study exploring this same procedure (five treatment patients and five control patients) noted no significant difference between opioid use in these two groups, but did document a shorter hospital stay for adolescents in the self-hypnosis group [95]. Numerous uncontrolled trials report results that trend in a positive direction including additional trials of hypnosis for abdominal pain, cancer, headaches, and juvenile classic migraine [106,107,108,109].

National training for pediatric hypnosis exists to teach providers across disciplines (e.g., physicians, psychologists, nurses, child life specialists) to use hypnosis tools and integrate them into clinical practice. In-person provider training with a child and adolescent focus is recommended before implementing this modality. Notably, in many geographic locations, patients may not have access to a provider trained in pediatric hypnosis.

#### 3.3.2. Hypnosis Summary

Like mindfulness-based interventions, hypnosis is a well-established integrative non-pharmacological intervention for chronic pain management among adults [100], and there is a smaller but growing research base for pediatric pain management and hypnosis [103,104]. While preliminary evidence is promising, more research will be necessary before providers can confidently assert that hypnosis aides in pain management in pediatric populations.

### 3.4. Acupuncture

Acupuncture is an ancient medical procedure that has been practiced in China and other East Asian countries for over 2000 years. The technique involves the placement of small needles at various locations in the body. Traditional explanations suggest that acupuncture works by activating and engaging movement in the body’s own energy resources, termed “Qi” [110]. Traditionally, acupuncture has been employed for the prevention of disease, as well as an early intervention in emerging illness. As an adjunct to current medical practice, acupuncture is often employed late in the course of illness (e.g., to assist with pain, loss of function), as well as to support withdrawal from opioid therapy. Related therapies include electroacupuncture, acupressure, moxibustion (i.e., burning of an herb near an acupoint to create local warming), laser stimulation of acupoints, and non-invasive stimulation of acupoints utilizing a transcutaneous electrical nerve stimulator (TENS, referred to as TEAS).

Studies exploring the mechanisms of action involved in acupuncture to better understand how acupuncture can lead to improvements in symptoms such as pain and withdrawal have focused on several areas. First, fMRI studies have demonstrated that acupuncture can lead to changes in brain blood flow, including the activity of the default mode network (DMN), and normalization of activity in areas of the limbic system often referred to as the “pain matrix” (e.g., insula, anterior cingulate gyrus, prefrontal cortex) [47,111,112]. Other work has shown that acupuncture can stimulate endorphin release in the central nervous system [113]. Research has not elucidated exactly how the needling procedures lead to these changes in brain activity; however, one possible explanation suggests activation of the cholinergic anti-inflammatory pathway—a neural pathway that modulates the body’s immune response to injury [114,115].

#### 3.4.1. Evidence for Acupuncture and Pain Management

In 1997, the NIH conducted a Consensus Meeting, where studies of acupuncture were reviewed by an expert panel. It was determined that sufficient evidence was present to recommend acupuncture for the treatment of adult postoperative pain, postoperative dental pain, and nausea and vomiting [116]. Other promising indications included the treatment of addiction. Since that time, multiple clinical studies have demonstrated that acupuncture was efficacious in the treatment of chronic back pain, headaches, and osteoarthritis among adults [117,118,119].

Pediatric applications have centered on the use of acupuncture for both acute and chronic pain [120]. Of particular relevance, research has demonstrated that acupuncture can decrease acute postoperative pain among pediatric populations. In a prospective randomized controlled study, Lin and colleagues found that acupuncture led to a statistically significant reduction in pain and agitation (*p* < 0.001) among 60 children undergoing bilateral myringotomy and tympanostomy compared to a control condition [121]. Additionally, fewer youth required additional analgesia in the acupuncture versus control condition. In another randomized controlled trial, acupuncture led to significantly less pain (*p* < 0.001) following tonsillectomy compared to a sham acupuncture condition [122].

Acupuncture treatment may also be useful in reducing opioid-related withdrawal symptoms among pediatric populations. In a randomized controlled trial to treat Neonatal Abstinence Syndrome, acupuncture led to a shorter duration of morphine treatment (28 days vs. 39 days in the control group; *p* = 0.019) and hospital stay (35 vs. 50 days in the control group; *p* = 0.048) [123]. Another study demonstrated that a course of acupuncture was helpful in supporting infants wean off a high dose opioid infusion in the intensive care unit [43].

Several randomized studies have explored in more detail the effect of acupuncture on pediatric chronic pain. Gottschling and colleagues performed a randomized trial using laser acupuncture, a non-invasive type of acupuncture, with 43 children who suffered from either migraine or tension type headache. The mean number of headaches per month decreased by 6.4 days in the group which received acupuncture treatment, and by 1 day in the placebo group (*p* < 0.001). Secondary outcomes of headache severity likewise decreased and were statistically significant at all time points [124].

In a small randomized controlled trial comparing acupuncture with sham acupuncture, 14 adolescents with pelvic pain due to endometriosis experienced a 4.8 (SD 2.4) point reduction in pain (on an 11-point numeric rating scale) after 4 weeks, which differed significantly from the control group who experienced an average reduction of 1.4 (SD 2.1) (*p* = 0.004). Reduction in pain was found to persist six months post-intervention; however, after four weeks the differences were not clinically significant, suggesting continued acupuncture may be necessary for a more prolonged therapeutic effect [125].

#### 3.4.2. Acupuncture Summary

Acupuncture appears to be an effective integrative non-pharmacological therapy in the management of acute postoperative pain and neonatal abstinence syndrome, and there is some preliminary evidence for chronic pediatric pain. Additional basic and clinical research is needed to adequately characterize the mechanisms of acupuncture and clinical effects on pain and withdrawal symptoms in pediatric populations. Given that acupuncture has been shown to be a well-tolerated and acceptable intervention among youth, feasible to implement in clinics, and has a low side effect profile, it should be considered for incorporation into multidisciplinary analgesia for youth with acute and chronic pain.

## 4. Intensive Interdisciplinary Pediatric Pain Rehabilitative Programs

As described above, the primary goal of pediatric pain management is functional restoration in concert with the management of discomfort. When multimodal analgesia and multidisciplinary outpatient care have not successfully met these goals, some youth are referred to comprehensive interdisciplinary pain programs. Interdisciplinary care involves “multimodal treatment provided by a multidisciplinary team collaborating in assessment and treatment using a shared biopsychosocial model and goals” [11]. This interdisciplinary approach involves escalation of intensity and quantity of standard therapies to better meet the need for this vulnerable population.

Intensive interdisciplinary pain treatment (IIPT) programs are becoming a popular treatment choice for children with chronic pain who exhibit significant functional disability and are unable to progress in an outpatient setting or for those who lack the proper resources in their local environment. IIPT involves patient and family participation in either an inpatient or outpatient day hospital setting with coordinated interventions among at least three disciplines including pain or rehabilitation physicians, psychologists, and physical and occupational therapists [38]. Logan et al. performed a longitudinal case series of 56 patients ages 8 to 18 suffering from Complex Regional Pain Syndrome (CRPS) and showed that an IIPT program led to improvements in functional disability, pain symptoms, medication use, and emotional functioning (all *p* < 0.01) [126]. Simons et al. showed that IIPT was superior to outpatient treatment with significantly larger improvements in functional disability, pain-related fear, and readiness to change among children with chronic pain [127]. Of note, Bruce et al. demonstrated that patients in a three-week IIPT program who were receiving opioids were able to be weaned off these medications and remained opioid-free at the 3-month follow-up [51]. While these results are promising, these studies require further investigation and corroboration. In the United States, 11 pediatric pain centers exist that offer this high level of coordinated treatment, varying in treatment duration (2–12 weeks) and daily treatment components [128].

## 5. Discussion

This article provides an overview of a multidisciplinary pain management approach for pediatric patients with acute and chronic pain, and highlights the evidence base of commonly utilized integrative non-pharmacological therapies for the treating pediatrician, primary health care pediatric provider, or non-pain pediatric specialist. The review explores a topic that is often overlooked in the medical literature, resulting in a noteworthy gap of knowledge among many providers caring for pediatric patients with complex medical and pain conditions. As such, this review aims to further inform providers about multidisciplinary pain management and highlight the role of integrative non-pharmacological therapies in medically complex situations by summarizing and synthesizing relevant literature. Currently, many patients often receive a medical cocktail including opioids, benzodiazepines, gabapentinoids, and other medications for pain management. The early incorporation of the integrative non-pharmacological therapies reviewed in this paper may help in lowering the doses of medications needed to attain comfort for this young and vulnerable population. Of note, there is preliminary evidence that some of the integrative non-pharmacological therapies reviewed, specifically hypnosis and acupuncture, may decrease the need for opioid therapy in inpatient populations (e.g., Nuss procedure, neonatal abstinence syndrome). While additional research is needed to explore the efficacy and generalizability of these results, these studies are encouraging and suggest that practitioners should specifically consider incorporating multidisciplinary pain management strategies where possible and feasible for patients for whom prolonged opioid therapy is being considered or prescribed for acute or chronic pain.

Additionally, many of the integrative non-pharmacological therapies reviewed consist of teaching skills that are applicable beyond an individual pain episode. The patient is taught to implement pain management skills themselves, which may increase the patient’s personal efficacy, coping resources, and overall resilience. These integrative non-pharmacological therapies can provide effective pain relief and give patients the feeling that they are able to have some “control” over a difficult pain management problem. Through increased knowledge and familiarity with these therapies and multidisciplinary pain management, the general pediatric practitioner is better equipped to help pediatric patients feel empowered to explore evidence-based integrative non-pharmacological options, creating an integrative process of care that is holistic, safe, and effective.

### Limitations

Although this paper is not a formal systematic review, it provides a thorough review of the literature that may be most pertinent to our intended audience—the general pediatric practitioner. The data base for the therapies described above in pediatric pain is still in its infancy, and therefore, few RCT’s exist within several the integrative non-pharmacological topic areas described (see Table A1). In addition, the heterogeneity of study population and design precludes a systematic comparison that would be of value to the general reader. In some instances, there was no pediatric data available on a particular topic. While mechanisms of action may differ between adult and pediatric populations due to developmental effects, adult data was reviewed where it was available and felt to contribute to an understanding of a mechanism of action of the therapy.

## 6. Conclusions

Multidisciplinary pain management, including pharmacological and integrative non-pharmacological therapies, has been demonstrated to be efficacious in the treatment of both acute and chronic pain. Pharmacological interventions include opioids and opioid-sparing agents that target specific aspects of the nociceptive and neuropathic pain physiology. Simultaneously, integrative non-pharmacological interventions such as CBT, MBIs, hypnosis, and acupuncture target the cognitive-affective and physiologic components of the pain experience, and support the cultivation of coping tools that can lead to long-term improvements in pain, psychological functioning, and quality of life. Given extant research, the incorporation of multidisciplinary pain management treatment including both multimodal pharmacological and integrative non-pharmacological therapies is recommended early in the process of caring for youth experiencing acute and chronic pain.

Suggested future directions include performance of larger pediatric trials across integrative non-pharmacological pain interventions, assessing the potential synergistic nature of combined integrative non-pharmacological therapies, and conducting further research on the transition from acute to chronic pain management. It is also recommended that studies more directly explore the associations and effects of multidisciplinary treatment on the necessity for and use of opioid therapies in pediatric pain, especially in chronic pain management, and on the possible utility of these therapies in weaning from opioids and other therapies. Overall, additional societal resources are urgently needed to increase availability of multidisciplinary pain management services to all youth impacted by acute and chronic pain.

## Figures and Tables

**Table 1:** Search data for pediatric pain articles and selected integrative non-pharmacological therapies (2000–2018).

Modalities	Ovid	Embase	Prospero	Cochrane	Pubmed	Number of Articles Screened	Number of Articles Reviewed
CBT	42	537	2	2	274	48	12
Mindfulness	37	53	2	17	26	45	25
Hypnosis	164	272	1	1	152	28	14
Acupuncture	149	357	2	24	132	32	14
Intensive Rehab	189	164	2	51	87	15	6
Multidisciplinary	324	1232	1	19	546	52	13

CBT = Cognitive Behavioral Therapy.

## Appendix A

**Table A1:** Selection of important studies in pediatric integrative non-pharmacological therapies reviewed for this paper. From all the articles screened, the articles with the highest level of evidence are summarized below.

Therapy Type	Authors (Reference Number)	Study Type (Grade of Evidence)	Study Population	Outcome	Key Results
CBT	Eccleston et al. [37]	Cochrane systematic review (1)	Cochrane systematic review of psychological therapies (37 RCTs; 2111 participants, mean age 12.45, SD 2.2)	Pain intensity; Disability	Reduced headache pain (RR 2.47, CI 1.97–3.09, *p* < 0.01; Reduced disability in headache pain (SMD −0.49, CI −0.74 to −0.24, *p* < 0.01); Reduced nonheadache pain (SMD −0.57, CI −0.86 to −0.27, *p* < 0.01); Reduced disability in nonheadache pain (SMD −0.45, CI −0.71 to −0.19, *p* < 0.01
	Palermo et al. [61]	Meta-analysis (1)	Meta-analysis (25 RCTs; 1247 participants, 9–17 yo)	Pain intensity	Decreased headache (OR 6.1, CI 4.06 to 9.15, *p* < 0.0001); Decreased abdominal pain (OR 7.5, CI 3.29 to 17.16, *p* < 0.001)
	Fisher et al. [62]	Systematic review and meta-analysis (1)	Meta-analysis (37 RCTs; 1005 participants, mean age 9.4, SD 1.14)	Pain intensity; Disability	Decreased non-headache pain (SMD −0.60, CI −0.91to −0.29, *p* < 0.001); Decreased nonheadache pain disability (SMD −0.27, CI −0.46 to −0.08, *p* < 0.01); Decreased headache (RR 2.9, CI 2.25 to 3.73, *p* < 0.001); Decreased headache at follow-up (RR 3.34, CI 2.02 to 5.53, *p* < 0.001
Mindfulness	Ruskin et al. [82]	Prospective pre-post interventional study (2)	21 adolescents, 12–18 yo with chronic pain	Feasibility; Acceptability	90.5% treatment completion rate; No dropouts; Compliance with home practice (*M* = 60 min/week); Would recommend group to a friend
	Chadi et al. [83]	RCT (2)	19 adolescents, 13.9–17.8 yo with chronic pain	Quality of life; Depression; Anxiety; Pain perception; Psychological distress; Salivary cortisol	No significant changes in quality of life, depression, anxiety, pain perception, or psychological distress; Cortisol levels decreased from an average of 3.37 (±1.72) pre-intervention nmol/L to 1.95 (±1.13) nmol/L post-intervention; Cohen’s d = 0.77, *p* < 0.001).
	Ali et al. [84]	Pilot (3)	15 adolescents, 10–18 yo with fibromyalgia, chronic fatigue, musculoskeletal pain, headache, or abdominal pain	Functional disability; Fibromyalgia symptoms; Quality of life; Mindfulness	Decreased functional disability (33% improvement, *p* = 0.026); improved fibromyalgia symptoms (26% improvement, *p* = 0.03), and improved mindfulness (child: 12% improvement, *p* = 0.02; parent: 17% improvement, *p* = 0.03).
	Waelde et al. [85]	Pilot (3)	20 adolescents, 13–17 yo with chronic pain	Feasibility and Acceptability Parental assessment	Feasible and acceptable; Decreased parental worry about child (*p* < 0.01)
	Hesse et al. [86]	Pilot (3)	20 adolescent females, 11–16 yo, with recurrent headaches	Quality of life; Pain acceptance; Depression	Improved quality of life, Parent assess PedsQL, *p* = 0.049; Improved child acceptance of pain, CPAQ-A *p* = −0.005; Improved child depression, CES-DC *p* = 0.009
Hypnosis	Manworren et al. [35]	RCT (2)	24 adolescents, 10–18 yo undergoing Nuss procedure	Pain intensity; Morphine equivalent/hour; Length of stay (LOS)	Lower mean pain intensity (−1.72, 95% CI 2.89–0.55, *p* = 0.0047); Lower maximum pain intensity (−2.27, 95% CI 3.73–0.82, *p* = 0.0028); Less morphine equivalents/hour (−0.13, 95% CI −0.25–0.00, *p* = 0.046); Shorter LOS (*p* = 0.0013)
Acupuncture	Tsao et al. [122]	RCT (2)	59 children, 3–12 yo undergoing tonsillectomy	Pain, Oral food intake	Improvement in pain control and oral intake (*p* = 0.0065 and 0.001, respectively)
	Raith et al. [123]	RCT (2)	28 newborns with neonatal abstinence syndrome	Duration of morphine treatment; Length of stay	Shorter duration of morphine treatment (28 days vs. 39 days; *p* = 0.019) and hospital stay (35 vs. 50 days; *p* = 0.048)
Rehabilitation	Hechler, et al. [38]	Systematic review (1)	10 studies (1020 adolescents, mean age 13.9, SD 1.5)	Pain intensity; Pain-related disability	Decreased pain intensity (d = −1.33, CI −2.28 to −0.38, *p* = 0.01); Decreased pain related disability (d = −1.09, CI −1.71 to −0.48, *p* < 0.001)
	Logan et al. [126]	Longitudinal case series (3)	56 children and adolescents, 8–18 yo with CRPS	Pain intensity; Functional disability; Subjective report of limb function, timed running, occupational performance, medication use, use of assistive devices, emotional functioning, anxiety and depression	Statistically significant improvements from admission to discharge in pain intensity, functional disability, subjective report of limb function, timed running, occupational performance, medication use, use of assistive devices, emotional functioning, anxiety, and depression. (all *p* < 0.01). (multiple outcomes)
	Simons et al. [127]	Comparative study (case-controlled) (3)	100 children and adolescents, mean age 13.9 (SD 2.17)	Functional disability; Fear of pain; Readiness to change	Improved functional disability (*p* = 0.00); Improved fear of pain (*p* = 0.00); Increased readiness to change (precontemplation *p* = 0.01, action/maintenance *p* = 0.00) (multiple outcomes)
	Bruce et al. [51]	Prospective longitudinal case series (3)	171 adolescents 12–18, mean age 15.3 (SD 1.73)	Functional disability; depressive symptoms; catastrophizing; Changes in opioid medication	Improved functional disability (*p* < 0.001) Improved depressive symptoms (*p* < 0.0001) Improved catastrophizing (*p* < 0.0001) Reduced pain severity (*p* = 0.0001) Decreased opioid medication (Out of 30 patients on opioids, 21 were weaned off medication, 1 did not finish program)

Notes: RCT, randomized controlled trials; SD, standard deviation; RR, risk ratio; CI, confidence interval; SMD, standardized mean difference; yo, years old; OR, odds ratios; M, mean; PedsQL, Pediatric Quality of Life Inventory; CPAQ-A, Chronic Pain Acceptance Questionnaire-Activity Engagement; CES-DC, Center for Epidemiological Studies of Depression Scale for Children; LOS, length of stay; d, effect sizes; CRPS, complex regional pain syndrome.

## References

[1] Pain terms: A list with definitions and notes on usage (1979). Recommended by the IASP Subcommittee on Taxonomy. Pain.

[2] Anwar K. (2016). Pathophysiology of pain. Dis. Mon..

[3] IASP Pain Terminology International Association for the Study of Pain Committee on Taxonomy. http://www.iasp-pain.org/Taxonomy#Pain.

[4] Slater M.E., De Lima J., Campbell K., Lane L., Collins J. (2010). Opioids for the management of severe chronic nonmalignant pain in children: A retrospective 1-year practice survey in a children’s hospital. Pain Med..

[5] Berde C., Nurko S. (2008). Opioid side effects—Mechanism-based therapy. N. Engl. J. Med..

[6] Moore R.A., McQuay H.J. (2005). Prevalence of opioid adverse events in chronic non-malignant pain: Systematic review of randomised trials of oral opioids. Arthritis Res. Ther..

[7] Gomes T., Mamdani M.M., Dhalla I.A., Paterson J.M., Juurlink D.N. (2011). Opioid dose and drug-related mortality in patients with nonmalignant pain. Arch. Intern. Med..

[8] Grunkemeier D.M., Cassara J.E., Dalton C.B., Drossman D.A. (2007). The narcotic bowel syndrome: Clinical features, pathophysiology, and management. Clin. Gastroenterol. Hepatol..

[9] Fudin J., Raouf M., Wegrzyn E.L. (2017). Opioid Dosing Policy: Pharmacological Considerations Regarding Equianalgesic Dosing.

[10] Lee M., Silverman S.M., Hansen H., Patel V.B., Manchikanti L. (2011). A comprehensive review of opioid-induced hyperalgesia. Pain Physician.

[11] Task Force on Multimodal Pain Treatment Defines Terms for Chronic Pain Care. http://www.iasp-pain.org/PublicationsNews/NewsDetail.aspx?ItemNumber=6981.

[12] Friedrichsdorf S.J. (2016). Contemporary pediatric palliative care: Myths and barriers to integration into clinical care. Curr. Pediatr. Rev..

[13] Dowell D., Haegerich T.M., Chou R. (2016). CDC guideline for prescribing opioids for chronic pain—United States, 2016. MMWR Recomm. Rep..

[14] Dash G.F., Wilson A.C., Morasco B.J., Feldstein Ewing S.W. (2018). A Model of the Intersection of Pain and Opioid Misuse in Children and Adolescents. Clin. Psychol. Sci..

[15] Schechter N.L., Walco G.A. (2016). The Potential Impact on Children of the CDC Guideline for Prescribing Opioids for Chronic Pain: Above All, Do No Harm. JAMA Pediatr..

[16] Friedrichsdorf S.J., Giordano J., Desai Dakoji K., Warmuth A., Daughtry C., Schulz C.A. (2016). Chronic Pain in Children and Adolescents: Diagnosis and Treatment of Primary Pain Disorders in Head, Abdomen, Muscles and Joints. Children (Basel).

[17] Chung C.P., Callahan S.T., Cooper W.O., Dupont W.D., Murray K.T., Franklin A.D., Hall K., Dudley J.A., Stein C.M., Ray W.A. (2018). Outpatient Opioid Prescriptions for Children and Opioid-Related Adverse Events. Pediatrics.

[18] Krane E.J., Weisman S.J., Walco G.A. (2018). The National Opioid Epidemic and the Risk of Outpatient Opioids in Children. Pediatrics.

[19] Lin Y.C., Lee A.C., Kemper K.J., Berde C.B. (2005). Use of complementary and alternative medicine in pediatric pain management service: A survey. Pain Med..

[20] Fisher E., Law E., Dudeney J., Palermo T.M., Stewart G., Eccleston C. (2018). Psychological therapies for the management of chronic and recurrent pain in children and adolescents. Cochrane Database Syst. Rev..

[21] Kamper S.J., Apeldoorn A.T., Chiarotto A., Smeets R.J., Ostelo R.W., Guzman J., van Tulder M.W. (2015). Multidisciplinary biopsychosocial rehabilitation for chronic low back pain: Cochrane systematic review and meta-analysis. BMJ.

[22] Lee C., Crawford C., Swann S. (2014). Active Self-Care Therapies for Pain (PACT) Working Group. Multimodal, integrative therapies for the self-management of chronic pain symptoms. Pain Med..

[23] Nicol A.L., Hurley R.W., Benzon H.T. (2017). Alternatives to opioids in the pharmacologic management of chronic pain syndromes: A narrative review of randomized, controlled, and blinded clinical trials. Anesth. Analg..

[24] Gardiner P., Lestoquoy A.S., Gergen-Barnett K., Penti B., White L.F., Saper R., Fredman L., Stillman S., Lily Negash N., Adelstein P. (2017). Design of the integrative medical group visits randomized control trial for underserved patients with chronic pain and depression. Contemp. Clin. Trials.

[25] Tick H., Nielsen A., Pelletier K.R., Bonakdar R., Simmons S., Glick R., Ratner E., Lemmon R.L., Wayne P., Zador V. (2018). Evidence-Based Nonpharmacologic Strategies for Comprehensive Pain Care: The Consortium Pain Task Force White Paper. Explore (NY).

[26] Waldman S. (2011). Pain Management.

[27] Odell S., Logan D.E. (2013). Pediatric pain management: The multidisciplinary approach. J. Pain Res..

[28] Gritsenko K., Khelemsky Y., Kaye A.D., Vadivelu N., Urman R.D. (2014). Multimodal therapy in perioperative analgesia. Best Pract. Res. Clin. Anaesthesiol..

[29] Brooks M.R., Golianu B. (2016). Perioperative management in children with chronic pain. Paediatr. Anaesth..

[30] Panella J.J. (2016). Preoperative care of children: Strategies from a child life perspective. AORN J..

[31] Suresh S., Wang S., Porfyris S., Kamasinski-Sol R., Steinhorn D.M. (2008). Massage therapy in outpatient pediatric chronic pain patients: Do they facilitate significant reductions in levels of distress, pain, tension, discomfort, and mood alterations?. Paediatr. Anaesth..

[32] Wang S.M., Escalera S., Lin E.C., Maranets I., Kain Z.N. (2008). Extra-1 acupressure for children undergoing anesthesia. Anesth. Analg..

[33] Brewer S., Gleditsch S.L., Syblik D., Tietjens M.E., Vacik H.W. (2006). Pediatric anxiety: Child life intervention in day surgery. J. Pediatr. Nurs..

[34] Yip P., Middleton P., Cyna A.M., Carlyle A.V. (2009). Non-pharmacological interventions for assisting the induction of anaesthesia in children. Cochrane Database Syst. Rev..

[35] Manworren R.C.B., Girard E., Verissimo A.M., Ruscher K.A., Santanelli J.P., Weiss R., Hight D. (2015). Hypnosis for postoperative pain management of thoracoscopic approach to repair pectus excavatum: Retrospective analysis. J. Pediatr. Surg. Nurs..

[36] Jensen B., Chen J., Furnish T., Wallace M. (2015). Medical marijuana and chronic pain: A review of basic science and clinical evidence. Curr. Pain Headache Rep..

[37] Eccleston C., Palermo T.M., Williams A.C., Lewandowski H.A., Morley S., Fisher E., Law E. (2014). Psychological therapies for the management of chronic and recurrent pain in children and adolescents. Cochrane Database Syst. Rev..

[38] Hechler T., Kanstrup M., Holley A.L., Simons L.E., Wicksell R., Hirschfeld G., Zernikow B. (2015). Systematic review on intensive interdisciplinary pain treatment of children with chronic pain. Pediatrics.

[39] Lee B.H., Scharff L., Sethna N.F., McCarthy C.F., Scott-Sutherland J., Shea A.M., Sullivan P., Meier P., Zurakowski D., Masek B.J. (2002). Physical therapy and cognitive-behavioral treatment for complex regional pain syndromes. J. Pediatr..

[40] Kuttner L. (2012). Pediatric hypnosis: Pre-, peri-, and post-anesthesia. Paediatr. Anaesth..

[41] Garland E.L. (2014). Disrupting the downward spiral of chronic pain and opioid addiction with mindfulness-oriented recovery enhancement: A review of clinical outcomes and neurocognitive targets. J. Pain Palliat. Care Pharmacother..

[42] Agoston A.M., Sieberg C.B. (2016). Nonpharmacologic treatment of pain. Semin. Pediatr. Neurol..

[43] Golianu B., Seybold J., Almgren C. (2014). Acupucture helps reduce need for sedative medications in neonates and infants undergoing treatment in the intensive care unit. Med. Acupunct..

[44] Schmitt Y.S., Hoffman H.G., Blough D.K., Patterson D.R., Jensen M.P., Soltani M., Carrougher G.J., Nakamura D., Sharar S.R. (2011). A randomized, controlled trial of immersive virtual reality analgesia, during physical therapy for pediatric burns. Burns.

[45] Brown M.L., Rojas E., Gouda S. (2017). A Mind-body approach to pediatric pain management. Children (Basel).

[46] Rabin J., Brown M., Alexander S. (2017). Update in the treatment of chronic pain within pediatric patients. Curr. Probl. Pediatr. Adolesc. Health Care.

[47] Dhond R.P., Yeh C., Park K., Kettner N., Napadow V. (2008). Acupuncture modulates resting state connectivity in default and sensorimotor brain networks. Pain.

[48] Jiang H., White M.P., Greicius M.D., Waelde L.C., Spiegel D. (2017). Brain activity and functional connectivity associated with hypnosis. Cereb. Cortex.

[49] Kucyi A., Salomons T.V., Davis K.D. (2016). Cognitive behavioral training reverses the effect of pain exposure on brain network activity. Pain.

[50] Becker W.C., Dorflinger L., Edmond S.N., Islam L., Heapy A.A., Fraenkel L. (2017). Barriers and facilitators to use of non-pharmacological treatments in chronic pain. BMC Fam. Pract..

[51] Bruce B.K., Ale C.M., Harrison T.E., Bee S., Luedtke C., Geske J., Weiss K.E. (2017). Getting back to living: Further evidence for the efficacy of an interdisciplinary pediatric pain treatment program. Clin. J. Pain.

[52] Chambless D.L., Hollon S.D. (1998). Defining empirically supported therapies. J. Consult. Clin. Psychol..

[53] Butler A.C., Chapman J.E., Forman E.M., Beck A.T. (2006). The empirical status of cognitive-behavioral therapy: A review of meta-analyses. Clin. Psychol. Rev..

[54] Beck J.S. (2011). Cognitive Behavior Therapy: Basics and Beyond.

[55] Wicksell R.K., Greco L.A. (2008). Acceptance and commitment therapy for pediatric chronic pain. Acceptance and Mindfulness Treatments for Children and Adolescents: A Practitioner’s Guide.

[56] Grave J., Blissett J. (2004). Is cognitive behavior therapy developmentally appropriate for young children? A critical review of the evidence. Clin. Psychol. Rev..

[57] Seminowicz D.A., Shpaner M., Keaser M.L., Krauthamer G.M., Mantegna J., Dumas J.A., Newhouse P.A., Filippi C.G., Keefe F.J., Naylor M.R. (2013). Cognitive-behavioral therapy increases prefrontal cortex gray matter in patients with chronic pain. J. Pain.

[58] Jensen K.B., Kosek E., Wicksell R., Kemani M., Olsson G., Merle J.V., Kadetoff D., Ingvar M. (2012). Cognitive Behavioral Therapy increases pain-evoked activation of the prefrontal cortex in patients with fibromyalgia. Pain.

[59] Williams A.C., Eccleston C., Morley S. (2012). Psychological therapies for the management of chronic pain (excluding headache) in adults. Cochrane Database Syst. Rev..

[60] Coakley R., Wihak T. (2017). Evidence-based psychological interventions for the management of pediatric chronic pain: New directions in research and clinical practice. Children (Basel).

[61] Palermo T.M., Eccleston C., Lewandowski A.S., Williams A.C., Morley S. (2010). Randomized controlled trials of psychological therapies for management of chronic pain in children and adolescents: An updated meta-analytic review. Pain.

[62] Fisher E., Heathcote L., Palermo T.M., de C Williams A.C., Lau J., Eccleston C. (2014). Systematic review and meta-analysis of psychological therapies for children with chronic pain. J. Pediatr. Psychol..

[63] Palermo T.M., Wilson A.C., Peters M., Lewandowski A., Somhegyi H. (2009). Randomized controlled trial of an Internet-delivered family cognitive-behavioral therapy intervention for children and adolescents with chronic pain. Pain.

[64] Huestis S.E., Kao G., Dunn A., Hilliard A.T., Yoon I.A., Golianu B., Bhandari R.P. (2017). Multi-Family Pediatric Pain Group Therapy: Capturing Acceptance and Cultivating Change. Children (Basel).

[65] Coakley R., Wihak T., Kossowsky J., Iversen C., Donado C. (2018). The Comfort Ability Pain Management Workshop: A Preliminary, Nonrandomized Investigation of a Brief, Cognitive, Biobehavioral, and Parent Training Intervention for Pediatric Chronic Pain. J. Pediatr. Psychol..

[66] Kabat-Zinn J. (1994). Wherever You Go, There You Are.

[67] Black D.S., Slavich G.M. (2016). Mindfulness meditation and the immune system: A systematic review of randomized controlled trials. Ann. N. Y. Acad. Sci..

[68] Grossman P., Niemann L., Schmidt S., Walach H. (2004). Mindfulness-based stress reduction and health benefits. A meta-analysis. J. Psychosom. Res..

[69] Ahola Kohut S., Stinson J., Davies-Chalmers C., Ruskin D., van Wyk M. (2017). Mindfulness-based interventions in clinical samples of adolescents with chronic illness: A systematic review. J. Altern. Complement. Med..

[70] Baer R.A. (2003). Mindfulness training as a clinical intervention: A conceptual and empirical review. Clin. Psychol. Sci. Pract..

[71] Garland E.L., Froeliger B., Zeidan F., Partin K., Howard M.O. (2013). The downward spiral of chronic pain, prescription opioid misuse, and addiction: Cognitive, affective, and neuropsychopharmacologic pathways. Neurosci. Biobehav. Rev..

[72] Garland E.L., Gaylord S.A., Palsson O., Faurot K., Douglas Mann J., Whitehead W.E. (2012). Therapeutic mechanisms of a mindfulness-based treatment for IBS: Effects on visceral sensitivity, catastrophizing, and affective processing of pain sensations. J. Behav. Med..

[73] Holzel B.K., Lazar S.W., Gard T., Schuman-Olivier Z., Vago D.R., Ott U. (2011). How does mindfulness meditation work? Proposing mechanisms of action from a conceptual and neural perspective. Perspect. Psychol. Sci..

[74] Zeidan F., Martucci K.T., Kraft R.A., Gordon N.S., McHaffie J.G., Coghill R.C. (2011). Brain mechanisms supporting the modulation of pain by mindfulness meditation. J. Neurosci..

[75] Zeidan F., Vago D.R. (2016). Mindfulness meditation-based pain relief: A mechanistic account. Ann. N. Y. Acad. Sci..

[76] Wager T.D., Scott D.J., Zubieta J.K. (2007). Placebo effects on human mu-opioid activity during pain. Proc. Natl. Acad. Sci. USA.

[77] Sharon H., Maron-Katz A., Ben Simon E., Flusser Y., Hendler T., Tarrasch R., Brill S. (2016). Mindfulness meditation modulates pain through endogenous opioids. Am. J. Med..

[78] Zeidan F., Adler-Neal A.L., Wells R.E., Stagnaro E., May L.M., Eisenach J.C., McHaffie J.G., Coghill R.C. (2016). Mindfulness-meditation-based pain relief is not mediated by endogenous opioids. J. Neurosci..

[79] Veehof M.M., Trompetter H.R., Bohlmeijer E.T., Schreurs K.M. (2016). Acceptance- and mindfulness-based interventions for the treatment of chronic pain: A meta-analytic review. Cogn. Behav. Ther..

[80] Hilton L., Hempel S., Ewing B.A., Apaydin E., Xenakis L., Newberry S., Colaiaco B., Maher A.R., Shanman R.M., Sorbero M.E. (2017). Mindfulness meditation for chronic pain: Systematic review and meta-analysis. Ann. Behav. Med..

[81] Anheyer D., Haller H., Barth J., Lauche R., Dobos G., Cramer H. (2017). Mindfulness-based stress reduction for treating low back pain: A systematic review and meta-analysis. Ann. Intern. Med..

[82] Ruskin D.A., Gagnon M.M., Kohut S.A., Stinson J.N., Walker K.S. (2017). A mindfulness program adapted for adolescents with chronic pain: Feasibility, acceptability, and initial outcomes. Clin. J. Pain.

[83] Chadi N., McMahon A., Vadnais M., Malboeuf-Hurtubise C., Djemli A., Dobkin P.L., Lacroix J., Luu T.M., Haley N. (2016). Mindfulness-based intervention for female adolescents with chronic pain: A pilot randomized trial. J. Can. Acad. Child Adolesc. Psychiatry.

[84] Ali A., Weiss T.R., Dutton A., McKee D., Jones K.D., Kashikar-Zuck S., Silverman W.K., Shapiro E.D. (2017). Mindfulness-based stress reduction for adolescents with functional somatic syndromes: A pilot cohort study. J. Pediatr..

[85] Waelde L.C., Feinstein A.B., Bhandari R., Griffin A., Yoon I.A., Golianu B. (2017). A pilot study of mindfulness meditation for pediatric chronic pain. Children (Basel).

[86] Hesse T., Holmes L.G., Kennedy-Overfelt V., Kerr L.M., Giles L.L. (2015). Mindfulness-based intervention for adolescents with recurrent headaches: A pilot feasibility study. Evid. Based Complement. Alternat. Med..

[87] Jastrowski Mano K.E., Salamon K.S., Hainsworth K.R., Anderson Khan K.J., Ladwig R.J., Davies W.H., Weisman S.J. (2013). A randomized, controlled pilot study of mindfulness-based stress reduction for pediatric chronic pain. Altern. Ther. Health Med..

[88] Ruskin D., Lalloo C., Amaria K., Stinson J.N., Kewley E., Campbell F., Brown S.C., Jeavons M., McGrath P.A. (2014). Assessing pain intensity in children with chronic pain: Convergent and discriminant validity of the 0 to 10 numerical rating scale in clinical practice. Pain Res. Manag..

[89] Zoogman S., Goldberg S.B., Hoyt W.T., Miller L. (2015). Mindfulness interventions with youth: A meta-analysis. Mindfulness.

[90] Zenner C., Herrnleben-Kurz S., Walach H. (2014). Mindfulness-based interventions in schools-a systematic review and meta-analysis. Front. Psychol..

[91] Sibinga E.M., Webb L., Ghazarian S.R., Ellen J.M. (2016). School-Based Mindfulness Instruction: An RCT. Pediatrics.

[92] Mani M., Kavanagh D.J., Hides L., Stoyanov S.R. (2015). Review and Evaluation of Mindfulness-Based iPhone Apps. JMIR Mhealth Uhealth.

[93] Hilgard E.R. (1965). Hypnotic Susceptibility.

[94] Spiegel H., Spiegel D. (1987). Trance and Treatment: Clinical Uses of Hypnosis.

[95] Lobe T.E. (2006). Perioperative hypnosis reduces hospitalization in patients undergoing the Nuss procedure for pectus excavatum. J. Laparoendosc. Adv. Surg. Tech. A.

[96] Gruzelier J.H. (1998). A working model of the neurophysiology of hypnosis: A review of the evidence. Contemp. Hypn..

[97] Jensen M.P., Patterson D.R. (2014). Hypnotic approaches for chronic pain management: Clinical implications of recent research findings. Am. Psychol..

[98] Crawford H.J., Horton J.E., Harrington G.C., Vendemia J.M.C., Plantec M.B., Jung S., Shamro C., Downs J.H. (1998). Hypnotic analgesia (Disattending pain) impacts neuronal network activation: An fMRI study of noxious somatosensory TENS stimuli. Neuroimage.

[99] Rainville P., Duncan G.H., Price D.D., Carrier B., Bushnell M.C. (1997). Pain affect encoded in human anterior cingulate but not somatosensory cortex. Science.

[100] Montgomery G.H., DuHamel K.N., Redd W.H. (2000). A meta-analysis of hypnotically induced analgesia: How effective is hypnosis?. Int. J. Clin. Exp. Hypn..

[101] Adachi T., Fujino H., Nakae A., Mashimo T., Sasaki J. (2014). A meta-analysis of hypnosis for chronic pain problems: A comparison between hypnosis, standard care, and other psychological interventions. Int. J. Clin. Exp. Hypn..

[102] Lambert S.A. (1996). The effects of hypnosis/guided imagery on the postoperative course of children. J. Dev. Behav. Pediatr..

[103] Liossi C., Hatira P. (1999). Clinical hypnosis versus cognitive behavioral training for pain management with pediatric cancer patients undergoing bone marrow aspirations. Int. J. Clin. Exp. Hypn..

[104] Vlieger A.M., Menko-Frankenhuis C., Wolfkamp S.C., Tromp E., Benninga M.A. (2007). Hypnotherapy for children with functional abdominal pain or irritable bowel syndrome: A randomized controlled trial. Gastroenterology.

[105] Vlieger A.M., Rutten J.M., Govers A.M., Frankenhuis C., Benninga M.A. (2012). Long-term follow-up of gut-directed hypnotherapy vs. standard care in children with functional abdominal pain or irritable bowel syndrome. Am. J. Gastroenterol..

[106] Anbar R.D. (2001). Self-hypnosis for the treatment of functional abdominal pain in childhood. Clin. Pediatr. (Phila).

[107] Olness K. (1981). Imagery (self-hypnosis) as adjunct therapy in childhood cancer: Clinical experience with 25 patients. Am. J. Pediatr. Hematol. Oncol..

[108] Kohen D.P., Zajac R. (2007). Self-hypnosis training for headaches in children and adolescents. J. Pediatr..

[109] Olness K., MacDonald J.T., Uden D.L. (1987). Comparison of self-hypnosis and propranolol in the treatment of juvenile classic migraine. Pediatrics.

[110] Lin Y.C., Wan L., Jamison R.N. (2017). Using integrative medicine in pain management: An evaluation of current evidence. Anesth. Analg..

[111] Hui K.K., Liu J., Marina O., Napadow V., Haselgrove C., Kwong K.K., Kennedy D.N., Makris N. (2005). The integrated response of the human cerebro-cerebellar and limbic systems to acupuncture stimulation at ST 36 as evidenced by fMRI. Neuroimage.

[112] Napadow V., Kettner N., Liu J., Li M., Kwong K.K., Vangel M., Makris N., Audette J., Hui K.K. (2007). Hypothalamus and amygdala response to acupuncture stimuli in Carpal Tunnel Syndrome. Pain.

[113] Wang S.M., Kain Z.N., White P. (2008). Acupuncture analgesia: I. The scientific basis. Anesth. Analg..

[114] Oke S.L., Tracey K.J. (2009). The inflammatory reflex and the role of complementary and alternative medical therapies. Ann. N. Y. Acad. Sci..

[115] Chavan S.S., Tracey K.J. (2014). Regulating innate immunity with dopamine and electroacupuncture. Nat. Med..

[116] (1998). NIH consensus conference: Acupuncture. JAMA.

[117] Haake M., Muller H.H., Schade-Brittinger C., Basler H.D., Schafer H., Maier C., Endres H.G., Trampisch H.J., Molsberger A. (2007). German Acupuncture Trials (GERAC) for chronic low back pain: Randomized, multicenter, blinded, parallel-group trial with 3 groups. Arch. Intern. Med..

[118] Zhao L., Chen J., Li Y., Sun X., Chang X., Zheng H., Gong B., Huang Y., Yang M., Wu X. (2017). The long-term effect of acupuncture for migraine prophylaxis: A randomized clinical trial. JAMA Intern. Med..

[119] Berman B.M., Lao L., Langenberg P., Lee W.L., Gilpin A.M., Hochberg M.C. (2004). Effectiveness of acupuncture as adjunctive therapy in osteoarthritis of the knee: A randomized, controlled trial. Ann. Intern. Med.

[120] Golianu B., Yeh A.M., Brooks M. (2014). Acupuncture for pediatric pain. Children (Basel).

[121] Lin Y.C., Tassone R.F., Jahng S., Rahbar R., Holzman R.S., Zurakowski D., Sethna N.F. (2009). Acupuncture management of pain and emergence agitation in children after bilateral myringotomy and tympanostomy tube insertion. Paediatr. Anaesth..

[122] Tsao G.J., Messner A.H., Seybold J., Sayyid Z.N., Cheng A.G., Golianu B. (2015). Intraoperative acupuncture for posttonsillectomy pain: A randomized, double-blind, placebo-controlled trial. Laryngoscope.

[123] Raith W., Schmolzer G.M., Resch B., Reiterer F., Avian A., Koestenberger M., Urlesberger B. (2015). Laser acupuncture for neonatal abstinence syndrome: A randomized controlled trial. Pediatrics.

[124] Gottschling S., Meyer S., Gribova I., Distler L., Berrang J., Gortner L., Graf N., Shamdeen M.G. (2008). Laser acupuncture in children with headache: A double-blind, randomized, bicenter, placebo-controlled trial. Pain.

[125] Wayne P.M., Kerr C.E., Schnyer R.N., Legedza A.T., Savetsky-German J., Shields M.H., Buring J.E., Davis R.B., Conboy L.A., Highfield E. (2008). Japanese-style acupuncture for endometriosis-related pelvic pain in adolescents and young women: Results of a randomized sham-controlled trial. J. Pediatr. Adolesc. Gynecol..

[126] Logan D.E., Carpino E.A., Chiang G., Condon M., Firn E., Gaughan V.J., Hogan M., Leslie D.S., Olson K., Sager S. (2012). A day-hospital approach to treatment of pediatric complex regional pain syndrome: Initial functional outcomes. Clin. J. Pain.

[127] Simons L.E., Sieberg C.B., Pielech M., Conroy C., Logan D.E. (2013). What does it take? Comparing intensive rehabilitation to outpatient treatment for children with significant pain-related disability. J. Pediatr. Psychol..

[128] American Pain Society Pain in Infants, Children, and Adolescents SIG. http://americanpainsociety.org/get-involved/shared-interest-groups/pediatric-adolescent-pain.

